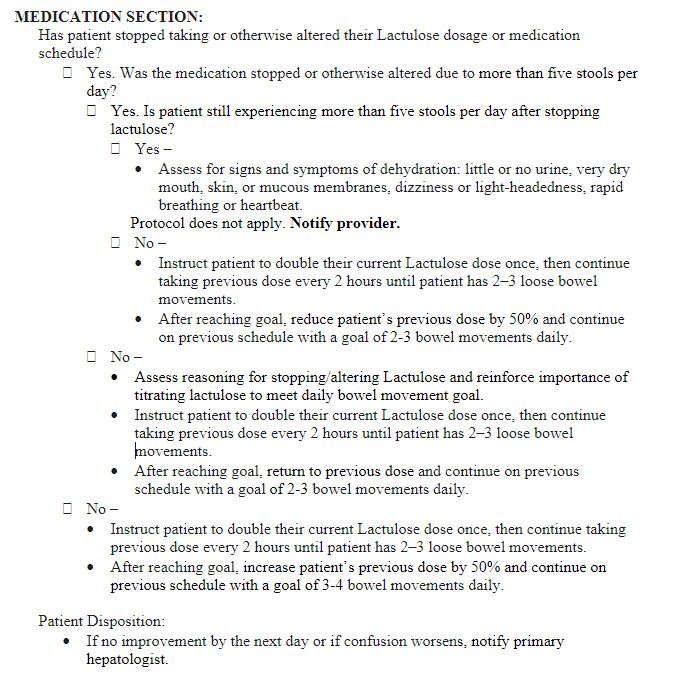# Design, implementation, and impact of a cirrhosis-specific remote patient monitoring program

**DOI:** 10.1097/HC9.0000000000000498

**Published:** 2024-07-22

**Authors:** Daniel D. Penrice, Kamalpreet S. Hara, Beatriz Sordi-Chara, Camille Kezer, Kathryn Schmidt, Blake Kassmeyer, Ryan Lennon, Jordan Rosedahl, Daniel Roellinger, Puru Rattan, Katherine Williams, Sara Kloft-Nelson, Angela Leuenberger, Patrick S. Kamath, Vijay H. Shah, Douglas A. Simonetto

**Affiliations:** 1 Division of Gastroenterology and Hepatology, Department of Internal Medicine, Mayo Clinic, Rochester, Minnesota, USA; 2 Department of Quantitative Health Sciences, Mayo Clinic, Rochester, Minnesota, USA; 3 Center for Connected Care, Mayo Clinic, Rochester, Minnesota, USA; 4 Center of Digital Health, Mayo Clinic, Rochester, Minnesota, USA

## Abstract

**Background:**

Remote patient monitoring (RPM) is an emerging focus in health care, and specialized programs may reduce medical costs, supplement in-office visits, and improve patient satisfaction. In this study, we describe the development, feasibility, and early outcomes of an RPM program for patients with decompensated cirrhosis.

**Methods:**

Forty-six patients were offered enrollment at the time of hospital discharge in the cirrhosis RPM program (CiRPM), of which 41 completed at least 30 days of monitoring. Participants were mailed remote monitoring equipment and a tablet to be used for patient-reported outcomes. Alerts were continuously monitored by virtual nursing staff who could perform targeted interventions. A cohort of historical controls (n = 74) was created for comparison using inverse probability of treatment weighting.

**Results:**

Patients were enrolled in the program for a mean of 83.9 days, with 28 (68%) completing the full 90-day program. Participants uploaded vital signs and responded to symptom-based questionnaires on 93% of the monitored days. On end-of-program surveys, over 75% of patients expressed satisfaction with the program. Gender, age, and MELD-Na were similar between CiRPM and weighted control groups. The 90-day readmission rate was 34% in CiRPM and 47% in weighted controls. In the CiRPM group, 12% of subjects had 2 or more admissions, compared to 37% in the weighted control group.

**Conclusion:**

This study demonstrates the feasibility of a cirrhosis-specific RPM program. Overall, patient satisfaction and utilization of the CiRPM was high. Future studies are needed to confirm the impact of RPM on the reduction of hospital readmissions in decompensated cirrhosis.

## Graphical abstract

**Figure:**
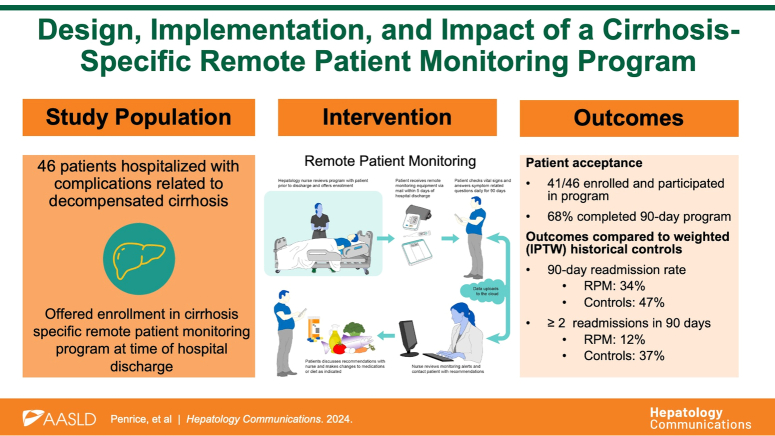

## INTRODUCTION

Cirrhosis affects more than 5 million patients in the United States and represents a major national cost, with more than 1.5 million hospitalizations and over $4 billion dollars in yearly health care spending.^1^ Readmission within 90 days of discharge in patients with cirrhosis has been shown to be as high as 53%, with 1-year mortality for those readmitted within 30 days exceeding 60%.^2^,^3^ Reasons for readmission vary widely, but complications of HE, ascites, variceal bleeding, infections, and renal/metabolic abnormalities are among the most frequently identified causes. The current standard of care in cirrhosis is one-time scheduled follow-up and/or phone communication with patients shortly after hospital discharge, which is only effective if problems have emerged before the visit/call. Additionally, as the prevalence of cirrhosis continues to increase nationwide, the health care system will likely be unable to sustain the current standard of care. Thus, novel solutions are urgently needed to address this gap.

The answer might be remote patient monitoring (RPM), an emerging area of focus in health care designed to increase surveillance of clinical events, decrease medical costs, supplement in-office visits, and increase patient and provider convenience. Kazanokov et al recently demonstrated the feasibility of a digital health program for managing patients with decompensated cirrhosis, finding that those enrolled in the program had fewer and shorter readmissions than matched controls.^4^ Other studies utilizing digital tools to manage patients with cirrhosis have shown the potential to avoid HE-related admissions, identify the need for paracentesis, and decrease complications after liver transplant.^5^,^6^,^7^

RPM is becoming increasingly popular, and its use will likely continue to increase in the near future due to recent changes in Medicare establishing a billing code for RPM.^8^ While studies have demonstrated promise in the use of digital tools to manage cirrhosis, it is essential to determine the feasibility and effectiveness of a comprehensive cirrhosis-specific RPM before widespread implementation of this technology.^9^,^10^,^11^ In this project, we aimed to: (1) design and implement a cirrhosis-specific RPM (CiRPM) program, as well as (2) show its utility through measurement of potential clinical benefit, patient satisfaction, and patient adherence. We hope this can serve as a roadmap for other personalized remote monitoring programs for patients with cirrhosis.

## METHODS

### Development of cirrhosis remote patient monitoring program (CiRPM):

The initial conception of a CiRPM program at our institution began in 2019. A program was envisioned to equip patients in their homes with Bluetooth-enabled sphygmomanometer, pulse oximeter, thermometer, and scale, along with a tablet with cellular connectivity to guide patients to measure vital signs and assess cirrhosis-specific symptoms daily while also facilitating educational content and video assessments by nursing staff, as needed. Any patient-reported data, whether actively collected or manually entered, would trigger alerts based on predefined criteria. Development was fast-tracked in late 2020 after the initial success of a COVID-19–specific remote monitoring program. Between June 2020 and April 2021, a multidisciplinary team was formed comprising leaders from the Center of Digital Health, physicians from the division of gastroenterology and hepatology, and hepatobiliary specialty nurses. The training was conducted with nursing teams on the enrollment of subjects. Additionally, the RPM nursing team was educated on the management of chronic liver disease and related complications and trained on targeted interventions such as titration of lactulose and diuretics.^12^

### *Implementation of* a *CiRPM program*

The CiRPM program was launched in April 2021. Patients hospitalized with complications of decompensated cirrhosis (HE, ascites, variceal bleeding, infection, jaundice, and/or renal/metabolic abnormalities) were contacted by hepatobiliary specialty nurses and enrolled within one day of hospital discharge. Subjects were identified through screening for ICD codes related to cirrhosis and then manually reviewed to assess for eligibility. Further eligibility criteria included age over 18 years, ability to provide written informed consent, and having established care with a gastroenterologist or hepatologist at our institution. Exclusion criteria for the program included residence in or discharge to a skilled nursing facility or rehabilitation unit, uncontrolled mental illness and/or drug or alcohol abuse without intention to quit, pregnancy, active cancer treatment, involvement in hospice care, and dementia or severe cognitive impairment. Patients who were identified as eligible were contacted by specialty hepatobiliary nurses within 1 day of dismissal from the hospital. During this visit, the program was explained, and the use of the technology was demonstrated.

Before discharge, patients were trained on the use of remote monitoring equipment. Upon discharge, enrolled patients were mailed the equipment as outlined above. Vital signs and PROs were automatically captured and uploaded into patients’ electronic health records in real-time without manual input (Supplemental Figure 1A, http://links.lww.com/HC9/A989). A dashboard containing all active patients in the program was created to facilitate monitoring by CiRPM nursing staff. Alert parameters, designed to identify patients at risk for clinical deterioration, were monitored 24/7 by the virtual nursing team who followed predefined decision trees. (Supplemental Figure 2, http://links.lww.com/HC9/A990 and 3, http://links.lww.com/HC9/A991) Program duration was 90 days.

**FIGURE 1:**
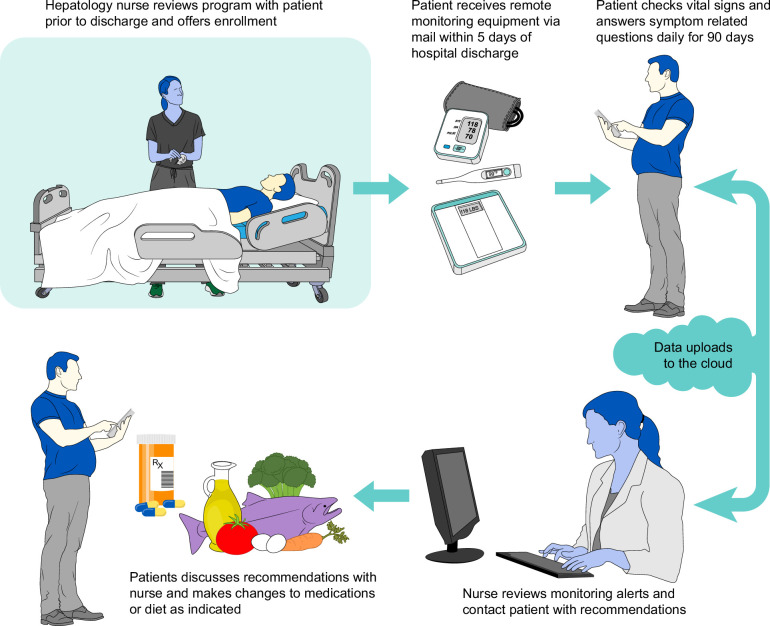
Visual representation of the CiRPM program. Abbreviation: CiRPM, cirrhosis remote patient monitoring program.

Six yes-no questions were delivered to patients’ tablets every 3 days (patients received 2 questions per day), including the following: (1) Have you, or someone close to you, noticed sudden changes in your attention, memory, speech, or thinking?; (2) Have you had new or worsening abdominal distention or bloating?; (3) Are you getting more short of breath more often?; (4) Do you have more swelling in your ankles than usual?; (5) Did you miss any doses of your medications this week?; (6) Are you having less than 3 soft bowel movements per day or more than 5 per day? A positive response to any of these questions would result in follow-up message or call from the RPM nursing team to discuss symptoms with the patient. The RPM nurse was empowered to perform interventions, including adjusting lactulose dose, arranging for paracentesis or clinic visit, and providing education. Participants also had the ability to directly message the RPM team to discuss specific concerns. For questions or symptoms outside the decision tree, the RPM nursing team contacted the primary gastroenterologist or hepatologist during business hours or the on-call provider after hours or weekends. RPM-generated data was stored within the patients’ electronic health record through a dashboard displaying vital signs and questionnaire responses (Supplemental Figure 1B, http://links.lww.com/HC9/A989, Figure 1).

### Study participants and comparison groups

**FIGURE 2:**
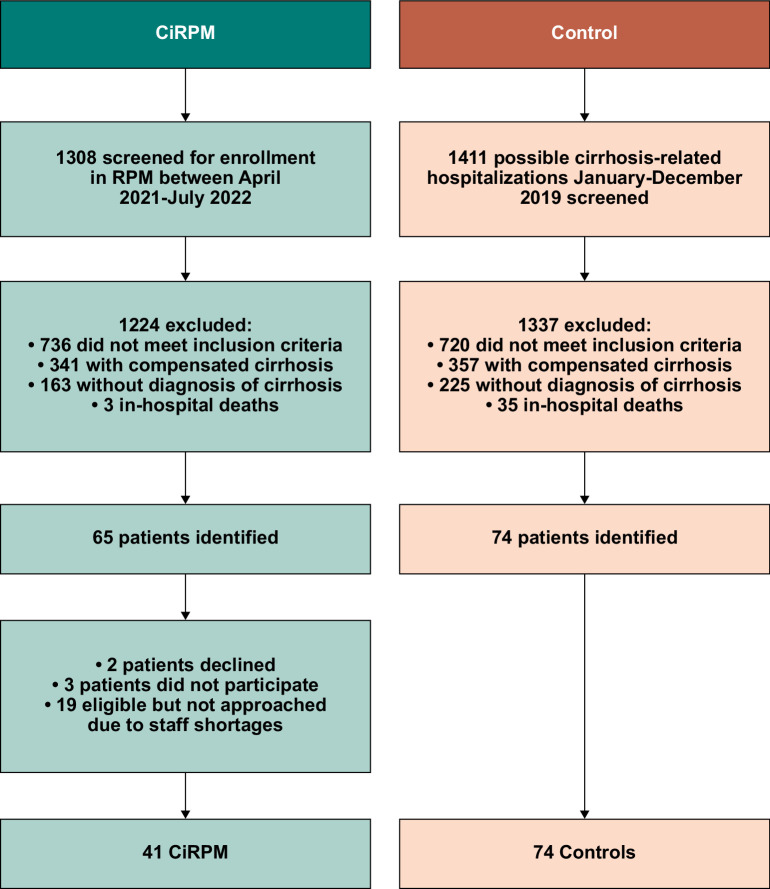
Creation of retrospective control cohort and CiRPM cohort. Abbreviations: CiRPM, cirrhosis remote patient monitoring program; RPM, remote patient monitoring.

From April 2021 through July 2022, 46 patients were offered enrollment in the RPM program, of which 44 agreed to participate and completed at least 30 days of monitoring. Two patients declined to participate and 3 were removed from the study after failing to upload vital signs after discharge. Of the 2 patients who declined, both expressed that the technology seemed too challenging to manage, and they did not feel they had the ability to participate. Of the 3 patients who initially agreed to be enrolled, 2 never uploaded any vital signs after discharge from the hospital and could not be reached by telephone. The other patient uploaded vitals for a few days after receiving the equipment but then decided they no longer wanted to participate. These 3 patients who initially agreed to participate but then did not engage with the program were not included in the final enrolled cohort. The control cohort included 74 patients identified during a retrospective review of consecutive cirrhosis-related admissions from January through December 2019. These controls met the inclusion criteria for CiRPM and likely would have been approached for enrollment had the program been active in 2019 (Figure 2).

### Statistical analysis

This study was conducted as a pilot feasibility study, and the primary focus was on the qualitative aspects of design, implementation, and patient satisfaction related to CiRPM. Our study was underpowered to detect differences in readmission and mortality.

Numeric variables were summarized using means and SDs. Categorical variables collected at the time of hospital admission were summarized using counts and percentages. Standardized differences were reported for weighted tables to assess balance. Controls were matched to patients enrolled in RPM using inverse probability of treatment weighting (IPTW) on demographic and patient characteristic variables. IPTW was employed to balance potential confounding variables while preserving the original sample size. IPTW weights were generated using logistic regression predicting treatment arm using patient characteristics and demographics until adequate balance was reached. Balance after weighting was assessed using balance tables and plots with the goal of obtaining standardized differences < 0.1 in key clinical characteristics. Before analyzing outcomes, balance was assessed by study personnel to ensure each arm had equivalent clinical risk. Thirty and 90-day readmissions were assessed using logistic regression. Ninety-day survival was assessed using a Kaplan-Meier curve. The number of readmission days in a 90-day window was modelled using a zero-inflated Poisson model. The RPM effect for the zero-inflated Poisson was tested using a likelihood ratio test. All models used the IPTW weights. All tests were two-tailed and conducted using a 0.05 threshold, and all CIs were constructed using a two-sided 95% CI. All analyses were performed in R 4.2.2 (R Foundation, Vienna, Austria).

## RESULTS

### Patient characteristics

The characteristics of enrolled individuals (N = 41) and retrospective controls (N = 74) after the inverse probability of treatment weighting are shown in Table 1. Enrolled and weighted control subjects were similar in age (median age 60.0 in each group) and race, with predominantly white individuals (93% and 94%, respectively). Gender was similar in enrolled and control individuals (42% vs. 46% female). The predominant cirrhosis etiology in the enrolled and control groups was alcohol (46% vs. 52%), followed by metabolic dysfunction–associated steatohepatitis (MASH) (27% vs. 30%). MELD-Na score at admission was also similar between both groups, with a median of 15.1 in CiRPM vs. 17.3 in control. Additionally, subjects who had a hospital admission in the 90 days before the index admission were also similar between the groups (27% in enrolled vs. 27% in control groups).

**TABLE 1:** Demographics and Clinical Characteristics of Subjects

Characteristics	RPM group n = 41	Retrospective controls n = 74	Standard difference
Age (y)	—	—	−0.16
* *Median (Q1, Q3)	60.0 (56.0, 67.0)	60.0 (56.0, 70.0)	—
* *Mean (SD)	60.9 (9.0)	62.4 (12.5)	—
Gender (n, %)	—	—	0.11
Female	17 (41.5)	34 (46.0)	—
Male	24 (58.5)	40 (54.0)	—
Etiology of liver Dx (n, %)			
* *Alcohol-associated	19 (46.3)	39 (52.3)	−0.02
MASH	11 (26.8)	22 (30.1)	−0.08
Other	11 (26.8)	13 (17.6)	0.11
Type of decompensation (n, %)			
* *Ascites	21 (51.2)	28 (38.5)	−0.13
* *HE	15 (36.7)	18 (24.3)	(−0.32, 0.24)
Variceal bleeding	5 (12.5)	2 (2.9)	−0.11
Jaundice	14 (35.0)	16 (22.6)	0.04
Infection	13 (32.5)	21 (28.9)	−0.09
* *Renal dysfunction	10 (24.4)	34 (47.1)	−0.29
Death			
* *Death in 90 d after admission	1 (2.4)	6 (7.7)	—
* *Death in the year after admission	9 (22.0)	21 (27.7)	—
Labs (median Q1, Q3)			
* *Sodium (mmol/L)	138.0 (133.0, 141.0)	136.0 (133.0, 140.0)	0.04
* *White blood cell count (× 10^9^/L)	6.4 (4.2, 9.4)	5.6 (4.3, 6.6)	0.25
* *Platelet count (× 10^9^/L)	103.0 (68.0, 126.0)	103.0 (74.2, 180.0)	−0.22
INR	1.5 (1.2, 1.9)	1.5 (1.3, 1.5)	0.13
Total Bilirubin	1.5 (0.8, 4.2)	1.2 (0.8, 6.8)	−0.04
Creatinine (µmol/L)	1.1 (0.8, 1.5)	1.1 (0.8, 1.7)	−0.2
Albumin (g/L)	2.9 (2.6, 3.4)	3.3 (3.0, 3.5)	−0.48
MELD-Na	—	—	−0.22
Median (Q1, Q3)	15.1 (9.8, 18.3)	17.3 (13.0, 18.42)	—
Mean (SD)	15.1 (9.6)	17.9 (8.3)	—
Charlson Comorbidity Index	—	—	−0.14
Median (Q1, Q3)	6.0 (5.0, 8.0)	6.0 (5.0, 8.0)	—
Mean (SD)	6.2 (2.0)	6.3 (2.1)	—
Baveno VII status			
First	7 (17.1)	16 (21.9)	−0.04
Further	30 (73.2)	51 (68.4)	0
CLIF-C AD Score			
* *Median (Q1, Q3)	53 (45, 63)	53 (46, 58)	0
Medications (n, %)	21 (51.2)	35 (47.7)	0.08
* *Lactulose	11 (26.8)	28 (37.7)	−0.24
Rifaximin	24 (58.5)	48 (64.5)	0.04
Furosemide	25 (61.0)	51 (68.6)	0.05
Sprionolactone	3 (7.3)	10 (13.5)	0.11
Other diuretic	6 (14.6)	7 (9.2)	0
Carvedilol	9 (22.0)	11 (14.7)	−0.05
Nadolol or propanolol	—	—	—

Abbreviations: CLIF-C AD, Chronic Liver Failure Consortium Acute Decompensation Score; MASH, metabolic dysfunction–associated steatohepatitis.

### Program utilization and patient satisfaction

A total of 46 patients were offered enrollment in the program, of which 41 participated in the program. Patients were enrolled in the program for a mean of 83.9 days (SD = 11.3). Due to the lack of caregivers at home and difficulty in arranging proper follow-up, 6 patients requested to remain on RPM longer than the 90-day monitoring period (maximum length was 124 d). However, for the purposes of this study, all data was capped at 90 days. A total of 28 (68%) patients completed the full 90-day program. When considering the 3 subjects who never engaged in the CiRPM, the program completion rate decreases to 64%. Of those who did not complete the full 90-day program,3 subjects dropped out between days 45 and 60, 2 dropped out between 60 and 75, and 8 dropped out between days 75 and 90. The most common reason for dropping out was patient preference due to clinical improvement and monitoring no longer deemed necessary. A few subjects ended monitoring early due to relocation or planned travel and did not wish to continue monitoring while traveling.

**FIGURE 3:**
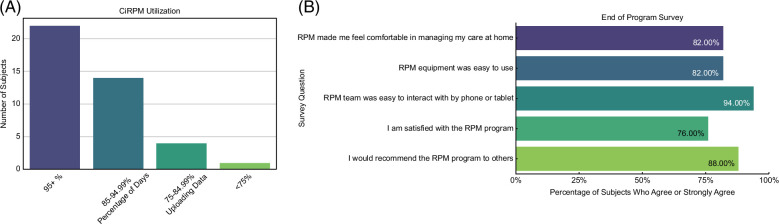
(A) Percentage of days participants in CiRPM uploaded data. (B) End-of-program questionnaire responses. Abbreviations: CiRPM, cirrhosis remote patient monitoring program; RPM, remote patient monitoring.

Participants uploaded vital signs and responded to questionnaires on 93% of the monitored days. We defined users as having high utilization if vital signs and PROs were completed on ≥ 85% of monitored days (excluding any days that the patient was hospitalized during the monitoring period). A total of 36 (88%) participants met this threshold of high utilization. Over half of the patients uploaded data on greater than 95% of monitored days (22/41, 54%). Only 1 participant uploaded data on less than 75% of days, and this was due to technology issues with reported inconsistent internet access (Figure 3A). When comparing high versus low utilization participants, no significant differences were seen with regard to the etiology of liver disease, insurance status, education level, alcohol use, or the presence of a caregiver at home.

Alerts were generated based on abnormal vital signs and PROs. There was an average of 189 (± 58) alerts generated per month. Of these alerts, an average of 6 (± 6) alerts per month required escalation to the primary hepatologist for further guidance on the next steps in management. Paracentesis was ordered based on alerts on 14 occasions.

A total of 17 participants filled out the end-of-program survey, with over 75% of respondents either agreeing or strongly agreeing with the following statements: CiRPM made me feel comfortable in managing my care at home; the CiRPM equipment was easy to use; the RPM team was easy to interact with by phone or tablet; I am satisfied with the CiRPM program; I would recommend the CiRPM program to others (Figure 3B).

### Hospital readmissions and mortality

**FIGURE 4:**
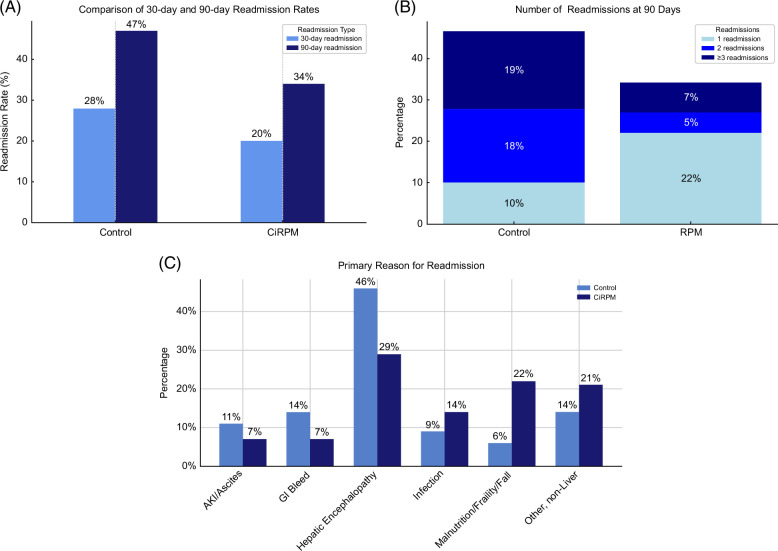
(A) 30 and 90-day readmission rates for Control and CiRPM cohorts. (B) Number of readmissions at 90 days for Control and CiRPM cohorts. (C) Reasons for readmission in Control and CiRPM cohorts. Abbreviations: AKI, acute kidney injury; CiRPM, cirrhosis remote patient monitoring program; RPM, remote patient monitoring.

The CiRPM group (N = 41) had nonsignificantly lower 30- and 90-day readmission rates compared to the control group. The 30-day readmission rate was 20% in the CiRPM group compared to 28% in the control group (OR: 0.46 [0.18, 1.97], *p* = 0.29. The 90-day readmission rate was 34% in the CiRPM group compared to 47% in the control group (OR: 0.59 [0.18, 1.93], *p* = 0.38) (Figure 4A).

**FIGURE 5:**
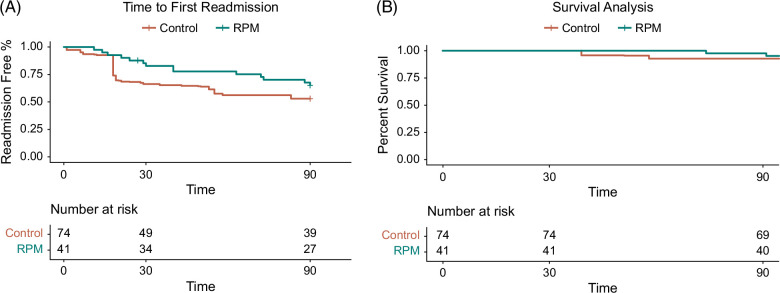
(A) Time to first readmission assessed using a Kaplan-Meier curve. (B) 90-day survival assessed using a Kaplan-Meier curve.

The median time to first readmission was 35 days in the CiRPM group compared to 18 days in the control group (*p* = 0.30) (Figure 5A). Total number of days hospitalized in the 90 days following discharge from index admission was lower in the CiRPM group (mean 3.4 hospital days) compared to the weighted control group (mean 4.9 d) (*p* = 0.067). There was also a trend towards fewer number of hospitalizations in the CiRPM group. In the CiRPM group, 12% of subjects had 2 or more readmissions, compared to 36.5% of subjects in the control group. (Figure 4B) Mortality in the 90 days postdischarge from index hospitalization was 2.4% in the CiRPM group and 7.7% in the control group (Figure 5B). At 1 year, 22% of those in the CiRPM group died compared to 28% in the control group. (HR 0.62 [0.20, 1.90]).

### Liver-related and potentially preventable readmissions

The CiRPM program had 41 patients who completed at least 30 days of the program. Reasons for readmission at 90 days were considered liver-related in 86% of the control cohort as compared to 79% of the CiRPM cohort (*p* = 0.63). The most common reasons for readmission in the control cohort were HE (45%), gastrointestinal bleeding (15%), and non-liver–related (15%). The most common reasons for readmission in the CiRPM cohort were HE (29%), malnutrition/frailty/falls (21%), and non-liver–related (21%) (Figure 4C).

In the CiRPM group, the rate of potentially preventable readmissions—defined as readmissions related to HE and fluid imbalance (acute kidney injury/ascites)—was 36% (5/14), whereas the control group experienced a higher rate of 57% (20/35) (*p* = 0.27). It should be noted that the concept of preventable readmissions is very subjective, and larger studies are needed to truly determine if remote monitoring can prevent these types of readmissions.^13^

### Remote management of HE

Early recognition of overt HE in patients with cirrhosis is crucial for prompt interventions, which may lead to a reduction in health care utilization. While enrolled in the CiRPM program, 21 (51%) patients were receiving lactulose and 11 (27%) were receiving Rifaximin or both. Five patients had HE-related readmissions while enrolled in CiRPM, all of which were preceded by vital sign alerts, and in 4/5 cases by self-reported confusion. Forty-four alerts for self-perceived confusion were reported by 15/41 patients, and 111 alerts for number of bowel movements outside goal range (2–4 BMs/d) were triggered in 19/41 patients. Nursing-initiated adjustments in lactulose dosing were made 39 times. As outlined above, the number of HE-related readmissions decreased from 45% in the control group to 29% in the CiRPM.

### Remote management of ascites

Similarly, early recognition of worsening ascites can prevent unnecessary hospitalizations or ED visits. While enrolled in the CiRPM program, 37 (90%) patients had ascites during index admission or were on diuretics due to a history of ascites. Fourteen of those patients had a total of 24 readmissions during which ascites was noted to be present. In the 2 weeks before readmission, 16/24 readmissions were preceded by reported symptom alerts, including abdominal distension, shortness of breath, and low compliance with required sodium intake. Despite 8 patients being noted to have ascites at the time of hospital readmission, ascites was not the primary reason for readmission in any of these cases. The median time from symptomatic alert to readmission was 2.5 days. Additionally, vital sign alerts such as low systolic blood pressure (< 90 mm Hg) or elevated heart rate (> 110 bpm) were triggered prior to 15/24 readmissions. Among these 15 readmissions,2 of the subjects were found to have SBP, which may have been a driver of these abnormal vital signs. A total of 78 outpatient paracenteses were done on 15 patients during the study period, with 49 /78 being preceded by vital signs or symptom alerts within the week before paracentesis. Nursing-initiated adjustments in diuretic dosing were made in response to alerts in 13/37 patients. All patients were encouraged to maintain a low sodium and high protein diet, and in cases where patients failed to meet dietary goals, they were re-educated.

## DISCUSSION

Hospital readmission among patients with decompensated cirrhosis is common and associated with increased mortality.^14^ The reasons for recurrent hospital visits encompass a wide spectrum of complications, such as HE, ascites, variceal bleeding, infections, falls, and renal/metabolic abnormalities.^15^ At present, the prevailing approach to cirrhosis care consists of a single, pre-scheduled follow-up appointment and occasional phone exchanges with patients shortly after their hospital discharge.^14^,^16^,^17^ However, this approach only proves effective if issues manifest before the scheduled visit or call. As the incidence of cirrhosis continues its upward trend on a national scale, the existing health care system may find it increasingly challenging to uphold this conventional standard of care.^18^ This predicament underscores an urgent need for innovative strategies and timely interventions.^19^

The rapid development and early success of remote monitoring programs to assist in managing the COVID-19 pandemic highlighted a novel approach to the management of who are acutely ill.^20^,^21^ In our study, we found that patients with decompensated cirrhosis were highly receptive to participating in an RPM program and were highly engaged with the program for the duration of monitoring. This is consistent with the recent study by Kazankov et al who reported that 85% (17 out of 20) of patients with advanced cirrhosis had a high engagement with their remote monitoring program.^4^ Other recent studies have demonstrated that high patient acceptance of smartphone applications for the management of cirrhosis and that high engagement with digital tools such as the patient portal is associated with decreased risk of hospital readmission.^22^,^23^ Our participants reported high satisfaction with the CiRPM program and felt that the program made them more comfortable in managing their care at home. Eighty-two percent of patients felt the RPM equipment was easy to use, and 88% would recommend the program to others.

For interventions after hospital discharge to be successful in reducing readmissions, we must be confident that at least some of them are potentially preventable. A study by Volk et al suggested that at least 22% of readmissions may be prevented. The most common causes of preventable rehospitalizations include HE and fluid imbalance, which can be caused by the inability to appropriately titrate lactulose, identify the need for paracentesis, or recognize the symptoms of over-diuresis.^13^ The potential for a decrease in up to 20% of cirrhosis-related readmissions represents a significant opportunity. Using ICD codes specific to cirrhosis, 90-day readmission rates for cirrhosis at our intuition over the past 20 years have ranged from 42% to 54%. There have been no trends in differences in readmission rates at our institution over this time period. In this study, we did observe a decrease in potentially preventable readmissions, with 57% of liver-related readmissions in the control group being secondary to HE or fluid imbalance (acute kidney injury/ascites), compared to only 36% in the CiRPM group. One of the first steps in developing an RPM program directed at patients with cirrhosis involves adequate training of or access to specialty nurses who are comfortable at titrating lactulose and diuretics, arranging for outpatient paracentesis, and referring patients to the clinic or the hospital when needed. Throughout the course of our study, abnormal vital signs and symptom-based queries alerted our RPM nursing staff to developing HE in 39 instances, which led to a targeted increase in lactulose dosing, potentially preventing related hospitalizations. Additionally, PROs and weight alerts triggered nursing staff to contact patients in 13 instances to discuss changes in diuretic dosing or to arrange outpatient paracentesis.

Our study attempts to address previously identified concerns with remote monitoring, such as variable patient adherence and disruptions in pre-existing workflows.^24^ Going forward, it is essential that health care institutions work towards removing potential barriers in access to technology. One of the main ethical issues of virtual and remote care is that it preferentially benefits those with higher incomes or those in metropolitan settings.^25^,^26^ Wireless internet available at home is currently a requirement for such digital tools, and patients with lower socioeconomic status or residing in rural areas might be less proficient with technology and, thus, be less willing to engage in its use.^27^ Thus, novel approaches are needed to help address these disparities.^28^,^29^,^30^

The primary strength of this study is the relatively large number of patients enrolled in the CiRPM program with high retention rates. Moreover, a historically matched cohort using inverse probability of treatment weighting allowed for comparisons on a number of health care utilization metrics without a reduction in our sample size. This study builds on the previously published CirrhoCare pilot by demonstrating the feasibility of a specialty-trained nurse-driven remote monitoring in a larger US-based population. The deliberate design and implementation of CiRPM, from patient identification and approach as part of the care continuum to automated real-time integration of remote data in the electronic medical records, will allow for rapid expansion of the program. The CiRPM program has recently been launched at Mayo Clinic, Arizona, with plans to extend to Mayo Health System hospitals and Mayo Clinic, Florida, in the near future. Although differences in outcomes were observed, the study was underpowered to reach statistical significance. As a pilot feasibility study, and due to the lack of adequately powered studies on the impact of RPM in cirrhosis, power calculations were not possible. Rather, our findings will serve as the basis for future adequately sized trials. Another limitation is the absence of cost analysis comparing total health care spending, which was not accessible due to institutional policies. Despite high usability and engagement, less than half (41%) of participants completed the end-of-program survey. A potential reason for this discrepancy is how and when surveys were delivered, as participants received the survey by email 4 weeks after the conclusion of the program. Additionally, the CiRPM program was provided at no cost to patients or insurance, which is currently not generalizable outside a research setting.

It is possible that readmission rates have changed between 2019 and the study period, regardless of the implementation of this program. However, there is some evidence that while rates of hospitalizations decreased during the COVID-19 pandemic, there has been an overall trend of increasing hospitalizations related to cirrhosis over the past decade.^18^ We specifically did not include the year 2020 due to the impact of the COVID-19 pandemic.

Another limitation is the use of a retrospective control group as opposed to a prospective control arm. Despite our best efforts to account for all variables with inverse probability weighting, there is a noted difference in the percentage of patients presenting with renal dysfunction in the control group. This could explain the increased rates of readmissions related to acute kidney injury and ascites in the control group.

This program involved 24/7 nursing monitoring so that if abnormal vitals were recorded at any time, they would be reviewed and, if needed, addressed by nursing staff. Although this represents a challenge, it is an important consideration from ethical and legal perspectives. With available monitoring equipment at home, patients may check vitals or send messages at any hour of the day or weekend. Furthermore, it would allow for the prevention of ED visits that may happen after hours. Larger studies will allow us to understand the magnitude of alerts and escalations outside business hours and help inform future cirrhosis RPM programs.

In summary, this study demonstrates that a RPM program designed to meet the unique needs of patients with cirrhosis is feasible and accepted by most patients. Initial findings suggest that such a cirrhosis-specific program may potentially lead to reduced hospital readmissions and decreased morbidity in this patient population. Now that feasibility has been established, larger studies are needed to determine the impact of RPM programs on cirrhosis.

## Supplementary Material

**Figure:**
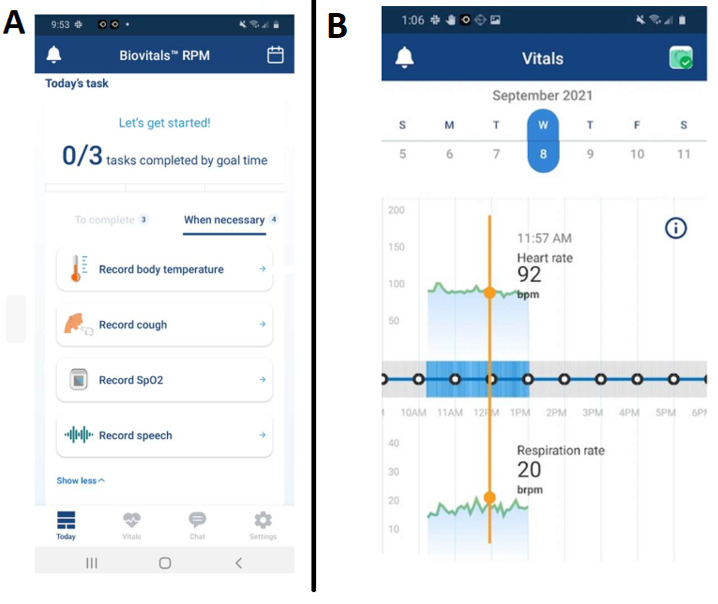


**Figure:**
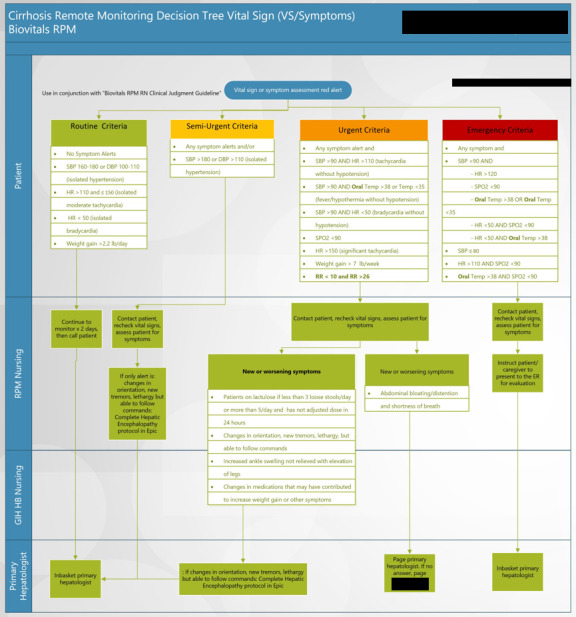


**Figure:**